# Integrating Health and Educational Perspectives to Promote Preschoolers’ Social and Emotional Learning: Development of a Multi-Faceted Program Using an Intervention Mapping Approach

**DOI:** 10.3390/ijerph17020575

**Published:** 2020-01-16

**Authors:** Claire Blewitt, Heather Morris, Kylie Jackson, Helen Barrett, Heidi Bergmeier, Amanda O’Connor, Aya Mousa, Andrea Nolan, Helen Skouteris

**Affiliations:** 1 Monash Centre for Health Research and Implementation, Monash University, Level 1, 43-51 Kanooka Grove, Clayton, Melbourne 3168, Australia; claire.blewitt@monash.edu (C.B.); heather.morris@monash.edu (H.M.); heidi.bergmeier@monash.edu (H.B.); mandy.oconnor@monash.edu (A.O.); aya.mousa@monash.edu (A.M.); 2 Bestchance Child Family Care, Melbourne 3150, Australia; kjackson@bestchance.org.au (K.J.); hbarrett@bestchance.org.au (H.B.); 3 School of Education, Faculty of Arts and Education, Deakin University, Geelong 3220, Australia; a.nolan@deakin.edu.au; 4 Warwick Business School, Warwick University, Coventry CV4 7AL, UK

**Keywords:** intervention mapping, intervention development, social and emotional learning, early childhood education and care, kindergarten, educator-child interactions

## Abstract

High-quality early childhood education and care (ECEC) can strengthen the social and emotional skills that are crucial for children’s ongoing development. With research highlighting an increasing prevalence of emotional and behavioural challenges in young children, there is emphasis on embedding teaching practices and pedagogies to support social and emotional skills within early learning programs. A growing body of research has examined the impact of social and emotional learning programs in ECEC; however, few studies describe the intervention development process, or how educators and other professionals were engaged to increase the relevance and feasibility of the program. The current paper describes the development of the Cheshire Social-Emotional Engagement and Development (SEED) Educational Program, an online learning tool to support early childhood educators to foster children’s positive mental health. Cheshire SEED was designed using five steps of the Intervention Mapping methodology: (i) comprehensive needs assessment to create a logic model of the problem; (ii) creation of program outcomes and change objectives mapped against determinants of educator behaviour; (iii) co-design of theory-based methods and practical strategies; (iv) program development; and (v) adoption and implementation planning. The process and decisions at each step of the IM protocol are presented, and the strengths and limitations of the approach to develop a mental health intervention for ECEC settings are discussed.

## 1. Introduction

Social and emotional competence in early childhood is an important predictor of ongoing health and wellbeing [1]. The cognitive and language abilities that emerge during this period support children to understand and regulate their emotion, attention, and behaviour, equipping them to form pro-social relationships and engage in learning [2,3,4]. For some preschoolers, however, difficulties in early social-emotional development can impair behaviour and functioning across family, school, and other settings [5,6,7], and predict long-term adverse outcomes including higher rates of mental and physical health problems, unemployment, substance abuse, and antisocial behaviour [8,9].

In addition to individual variables such as genetics, temperament, physical health and cognitive functioning, familial factors including financial disadvantage, parental mental health, low self-efficacy and stress, certain parenting styles, exposure to family violence, and insecure caregiver–child attachment histories [10,11,12,13,14,15,16,17,18,19,20,21], and racial oppression [22] have been associated with social-emotional maladjustment. Epigenetic research highlights the complex interplay between environmental factors and gene expression, and the effects on risk and protective pathways for children’s psychological development [23,24,25]. A global review of epidemiological studies suggests that between 9.5 and 14.2% of children aged five years and under experience serious emotional and behavioural disturbance [13]. In Australia, a national survey of the mental health and wellbeing of Australian children and adolescents found 13.6% of children aged four to eleven years met diagnostic criteria for a mental health disorder (encompassing anxiety disorder, major depressive disorder, attention deficit hyperactivity disorder and conduct disorder) [26]. Such findings highlight the importance of early intervention and prevention to improve short and long-term outcomes [8].

The potential to foster children’s social and emotional development through early childhood education and care (ECEC) programs has experienced a surge of attention from educators, policy-makers and researchers over recent decades [2,27]. A substantial body of research indicates that high-quality ECEC can improve the social, emotional and cognitive skills that are crucial for future learning and wellbeing [28,29,30,31,32], especially for children experiencing economic disadvantage [33,34,35]. The quality of interactions between educators and children are a vital component of service provision with regards to developmental outcomes [36,37,38,39]. However, studies suggest children attending ECEC services are not consistently exposed to the quality of interactions required for optimal development [36,40,41,42,43,44].

One way in which early learning programs can respond to children’s social and emotional needs is through social and emotional learning (SEL) intervention. SEL describes the active process whereby children attain and apply knowledge and skills relating to self-awareness, social awareness, self-management, relationships, and responsible decision-making [45]. Programs may include explicit lesson-based skill instruction, integrating SEL into existing pedagogy and curriculum, and practices embedded into daily interactions and the learning environment [46,47].

While a growing body of research has examined the impact of SEL programs in ECEC, few studies describe the intervention development process [48] or how educators and other professionals were engaged to increase the relevance and feasibility of programs. One possible solution to address this gap is to use Intervention Mapping (IM) to guide the development of new innovations. IM is a program planning, implementation, and evaluation framework, underpinned by theoretical and evidence-based decision making, a participatory-based research approach, and a systems-science perspective that explicitly addresses individual, interpersonal, community and societal influences on behaviour and health outcomes [49]. It has been applied extensively to design complex health-related behaviour change programs. More recently, researchers have suggested IM offers a promising framework to design interventions focused on supporting young children’s social and emotional development [48,50], yet, to our knowledge, no studies have described the use of IM in the context of SEL interventions, and there is a paucity of literature that provides detail and transparency regarding design processes.

In the current study, we sought to adapt and translate practices from an existing evidence-based educational program, called The Cheshire School, into the early years environment. The Cheshire School is an 18-month intervention program for children aged 4–11 years who experienced significant social, emotional, and behavioural challenges in mainstream school [51]. The resulting SEL program, the Cheshire Social-Emotional Engagement and Development Educational Program (Cheshire SEED), is a multi-faceted learning intervention for early childhood educators to build expertise and knowledge to foster children’s social and emotional skills, with strategies and techniques that can be embedded into everyday practice. As such, the aim of this paper is to describe the use of IM methodology to develop a SEL intervention to support ECEC educators to strengthen children’s positive mental health.

## 2. Method

The IM framework is a six-step iterative process, where each step builds on the decisions and products produced in the preceding steps [49]. The sections below summarise how the IM process was used to develop the Cheshire SEED Program, including: (i) logic model of the problem; (ii) program outcomes and objectives; (iii) program design; (iv) program production; and (v) program implementation plan. Step 6 of the protocol focuses on evaluation planning, which is outside the scope of this paper and will be reported in a subsequent paper.

### 2.1. Research Setting

This research project was conducted in Victoria, Australia. Regulation, assessment and quality improvement for Australian ECEC services is guided by the National Quality Standard [52], with early years services rated against seven quality areas: educational program and practice, children’s health and safety, physical environment, staffing arrangements, relationships with children, collaborative partnerships with families and communities, and governance and leadership. Childhood curriculum and pedagogy is also informed by Belonging, Being, Becoming—The Early Years Learning Framework (EYLF) [53]. This national framework is designed to encourage informed curriculum decisions, emphasising play-based learning, communication, language, and social and emotional development. The EYLF identifies five learning outcomes for children: children have a strong sense of identity, are connected with and contribute to their world, have a strong sense of wellbeing, are confident and involved learners, and effective communicators. Principles that underpin educator practice are also specified: secure, respectful and reciprocal relationships, partnerships, high expectations and equity, respect for diversity, and ongoing learning and reflective practice. Additionally, the framework highlights pedagogical practices that promote children’s learning, including adopting holistic approaches, responsivity to children, intentional teaching, creating physical and social learning environments, planning and implementing learning through play, and assessing and monitoring learning to support learning outcomes [53].

### 2.2. Step 1: Logic Model of the Problem

Step 1 considered the epidemiologic, behavioural, and social perspectives of the community at risk for health-related problems (preschool-aged children), the intervention target population (early childhood educators), and the program setting (ECEC services). A detailed assessment of the needs and capacity of early childhood educators with regards to supporting children’s social and emotional development was undertaken to inform the program logic and program goals.

#### 2.2.1. Intervention Design Group

An intervention design group was convened to provide input, guidance, and oversight of the development process. This group included eight participants: two educators, one with experience in early childhood settings, and the other with experience in both early childhood and primary programs, including The Cheshire School; three paediatric psychologists working within The Cheshire School, one of whom was also a play therapist; one ECEC pedagogical leader; and two researchers with expertise in developmental psychology. The group met regularly through the 18-month design period to discuss the enablers and barriers for educators in supporting children’s social-emotional development, findings from a series of literature reviews and qualitative research, intervention co-design, trialling and refining the program, program implementation, and evaluation. In addition, regular input was sought from individuals who could inform intervention design and delivery, including senior ECEC managers and a speech therapist.

#### 2.2.2. Literature Reviews

Pertinent literature was reviewed to understand the determinants of educator behaviour with regards to children’s social and emotional skills, and the availability and benefits of existing SEL programs for preschool-aged children. SEL intervention in educational settings have been described within a response-to-intervention tiered model, with intervention intensity aligned to child need [54,55]. Tier 1 programs are offered universally to all children as a proactive and preventative approach; Tier 2 interventions target select children experiencing, social, emotional, or behavioural challenges, who may not have responded to universal approaches; and Tier 3 supports are delivered to children requiring intensive and comprehensive assistance, who may display symptoms related to mental health disorders [56].

The response-to-intervention model was used as a framework to review the availability and outcomes associated with SEL programs, and to ensure that Cheshire SEED did not replicate, but added to, existing approaches. The following reviews were conducted: (i) systematic literature review and meta-analysis examining the social, emotional, and early learning outcomes associated with universal (Tier 1) curriculum-based SEL programs delivered to children aged 2–6 years in ECEC settings [57]; (ii) systematic literature review examining the effectiveness of universal (Tier 1) SEL programs on educator outcomes, including teaching quality and practice; (iii) systematic literature review examining the effectiveness of targeted (Tier 2) SEL programs on child outcomes [58]; and (iv) a narrative review to explore the breadth and benefits of educator-led Tier 3 SEL intervention delivered to children with mental health or developmental challenges in inclusive ECEC settings [59]. A targeted literature review (non-systematic) was used to explore the determinants of educator behaviour with regards to children’s social and emotional skills.

#### 2.2.3. Qualitative Interviews and Focus Groups

Semi-structured key informant interviews (*n* = 13 participants) and three focus groups (*n* = 17 participants) were conducted with professionals working within the ECEC sector. The key informant interviews were carried out by one author (Claire Blewitt). The three focus groups discussions were facilitated by two authors (Heidi Bergmeier and Helen Skouteris) and one researcher with a Doctorate in Psychology. Twenty educators (working in both kindergarten and full day childcare rooms) from four Melbourne-based ECEC centres took part, along with five staff who held a leadership or executive management position with oversight of ECEC service provision; three researchers with expertise in early child development within ECEC settings; and two staff from non-government agencies with knowledge or involvement in efforts to increase early social and emotional development. Questions were consistent across interviews and focus groups, and aimed to ascertain participant knowledge of children’s social and emotional development in early childhood, approaches or strategies to support children’s social and emotional development, enablers that support knowledge and skills, perceived barriers to SEL, and potential pathways to overcome these barriers. Interviews were audio-recorded and transcribed by one author (Claire Blewitt). Two authors (Claire Blewitt and Amanda O’Connor) coded 20% of transcripts to ensure the identification of consistent themes. Any discrepancies were resolved by discussion. One author (C.B.) then coded the remaining transcripts. Research themes were cross-checked with the research team to ensure accurate coding of participant perspectives. Thematic analysis [60] was used to identify patterns and descriptive topics reported by participants. The findings provided insight into the strategies and techniques ECEC educators employ to encourage children’s social and emotional development, and the barriers and opportunities for strengthening practice across the sector.

### 2.3. Step 2: Identification of Program Outcomes and Objectives

Step 2 focused on specifying detailed outcomes for the Cheshire SEED Program. Guided by the socio-ecological model [61], a behavioural outcome at the individual educator level, and environmental outcomes at the interpersonal and organisational levels were established. Drawing on information generated during Step 1, each outcome was then subdivided into performance objectives (explicit behaviours required to achieve each behavioural and environmental outcome). Matrices of change were created by cross-tabulating performance objectives with the determinants identified during Step 1 to create change objectives, that is the change needed in the determinant for educators to achieve the performance objective. A separate matrix was created for each intervention level.

### 2.4. Step 3: Program Design

In Step 3, the intervention was conceptualised and designed. Over three months, the intervention design group participated in three workshops (facilitated by Dialogic Learning [62] using the d.School Design Thinking Process) to generate broad ideas for program scope, themes, and delivery. Reflecting the iterative nature of the IM approach, participants revisited the outcomes from Steps 1 and 2, with a focus on empathising with the target population, mapping the connections between stakeholders in the early childhood sector, confirming the problem statement and program goal, and acknowledging any assumptions. Participants were encouraged to generate potential program ideas by workshopping the tools, skills, mind-set and processes needed to achieve the program outcomes and objectives. Ideas were then grouped into themes, discussed and prioritised. The design group created a number of prototypes and tested these with small groups of educators who would ultimately be the end-user of the intervention.

Following these workshops, one author (Claire Blewitt) identified theory and evidence-based behaviour change methods (general techniques for influencing determinants of the target group) and practical applications (specific activities to operationalise the theory-based methods) for the determinants and change objectives produced in Step 2. Methods and applications were aligned to the principles and pedagogical practices in the EYLF. Practical applications were then embedded into the program components identified during the design workshops.

### 2.5. Step 4: Program Production

During this phase, detailed program content and materials for Cheshire SEED were prepared. First, three members of the intervention design group (a Paediatric Psychologist/Play Therapist, Senior Cheshire Educator with experience in early years education, and researcher) mapped teaching practices and strategies that support young children’s social and emotional skill development. This process drew upon learning from the literature synthesis and qualitative research in Step 1, and the practice and evidence-informed knowledge of participants. Strategies were mapped against five challenging behaviours that can emerge in early childhood (anxious or withdrawn, oppositional, aggressive, hyperactive or impulsive, emotionally reactive) [63], in addition to universal strategies that can benefit all children, and the time of day that the strategy could be applied (e.g., arrivals, transitions, child-led play, or educator-led activities). Techniques were then prioritised and the detailed structure, content, and materials developed with the intervention design group.

The intervention design group proposed a pilot trial and feasibility evaluation of Cheshire SEED within two ECEC settings to assess and refine the program and delivery model. One ECEC service acted as a wait list control group; the full results of this pilot study will be reported once the pilot is completed. Educators from a kindergarten service (working with children aged three to five years) were invited: (i) to participate in a workshop that included reflection on the personal opportunities and challenges in their role and the strengths and challenges for children within their groups, an introduction to the Cheshire SEED intervention, including universal techniques for a “typical day”, strategies for working with children with challenging behaviour, and two case studies selected by participants; (ii) to access a preliminary version of the online portal that provided information on children’s social-emotional development, allowed educators to record their goals, and suggested evidence-based strategies focused on the learning environment and therapeutic educator–child interactions; and (iii) to access the final version of the program that included additional content and strategies. Intervention group educators also participated in two in-person consultation sessions with experts from the Cheshire School (the Paediatric Psychologist/Play Therapist and Senior Educator). During consultation sessions, the educator was observed in-session before meeting one-on-one to discuss their priorities and recommended strategies in detail. As part of the outcome evaluation, feedback was collected from educators following the completion of each component. In addition, educators were invited to complete surveys at baseline and again at the end of the implementation period to assess their perception of the quality of their relationships with children, and their self-efficacy and beliefs related to fostering social-emotional skills within the early learning environment. Educators were also interviewed to gather further insights regarding the social validity and feasibility of the program.

### 2.6. Step 5: Program Implementation Plan

Step 5 focused on the creation of an implementation plan to encourage adoption and maintenance of Cheshire SEED. The focus groups and interviews in Step 1, discussion with the intervention design group and other ECEC leaders informed our understanding of potential program users and how the intervention could be delivered to and embedded within early childhood services. Step 5 utilises a similar process to Step 2. Outcomes, performance objectives and determinants for program adoption and implementation were defined based on theory and evidence. A matrix of change objectives was created by linking performance objectives to determinants, and a plan for implementation created.

### 2.7. Step 6: Evaluation Plan

The final step of the IM process involves the design and implementation of an evaluation plan, which is out of the scope of the current paper.

## 3. Results

### 3.1. Step 1: Logic Model of the Problem

#### 3.1.1. Determinants of Educator Behaviour

Several personal attributes appear to influence educators’ ability to support children’s social and emotional development. Educator practice and decision-making is influenced by beliefs and experiences, in addition to theories studied during pre-service training and other learning opportunities that resonate with those beliefs and experiences [64]. High levels of self-efficacy are associated with positive expectations for children [65], increased use of high-quality practices in preschool rooms [66], and time spent teaching social, emotional, and cognitive skills [67]. Goroshit and Hen [68] reported high levels of emotional self-efficacy predicted empathy and teaching self-efficacy, both critical for positive teaching and child learning.

A related attribute is educator knowledge. SEL interventions use instructional processes (explicit or implicit) to strengthen children’s social-emotional health. Research shows greater content knowledge is related to improved pedagogical self-efficacy [69,70]. Finally, educators own social and emotional wellbeing influences their ability to support positive mental health in others. Educators with high self and social awareness understand and regulate their emotions, and recognise and effectively respond to emotions in others, thereby helping to build strong relationships and facilitate positive outcomes for children [71]. Conversely, preschool educator stress is associated with lower levels and less consistent emotional support [72], lower quality teaching practices, and lower quality communication with parents [67].

#### 3.1.2. Literature Reviews of SEL Programs

The literature reviews aimed to explore the availability of SEL programs across three tiers of intervention (universal, targeted and intensive), the benefits for children and educators, and the specific program components related to program success. The key findings from each study are summarised in Table 1. Most SEL programs were delivered at the class-wide level. Universal interventions appeared to benefit children across social, emotional, behavioural, and learning domains. Research evidence for programs that target children experiencing social, emotional, or behavioural difficulties is emerging; however, the studies reviewed suggest interventions predominately focus on children displaying externalising problems such as aggression or antisocial behaviour. Based on the studies captured, there are few evidence-based approaches for educators working with children with internalising challenges (e.g., anxiety and withdrawal).

#### 3.1.3. Qualitative Interviews and Focus Groups

Four themes emerged from the thematic analysis of qualitative interviews and focus group discussions: (i) educator knowledge—explicit and tacit dimensions; (ii) mobilising knowledge—social and emotional learning is embedded within interactions; (iii) room for improvement—capacity and capability; and (iv) strengthening educator skill—building knowledge through practical strategies. First, early childhood educators revealed explicit and tacit dimensions to their knowledge of children’s socioemotional health. Educators referred to a broad range of competencies to describe early social and emotional development, indicators of social and emotional challenges, and risk and protective factors relating to social-emotional development, reflecting their explicit knowledge. They also drew on tacit knowledge, formed through their own experiences in the classroom, working with children with diverse and individual needs, observation of their peers, and interaction, discussion, and reflection with colleagues and specialists.

Next, strategies to support children’s social-emotional skills were embedded within interactions. The educator–child relationship was unanimously acknowledged by both educators and non-classroom based early childhood professionals as critical to children’s development. Targeted strategies to support SEL were embedded within everyday experiences and interactions; however, there was variation in the breadth of strategies identified across participants. In addition, the influence of the layout and organisation of the preschool classroom was highlighted, with educators using physical resources and materials to encourage prosocial behaviour (e.g., re-directing children to preferred activities). The importance of working in partnership with caregivers was consistently highlighted.

Third, participants identified an extensive range of programs and resources to support social and emotional development within preschool settings. However, the volume of programs available and increasing expectations placed upon educators meant programmatic approaches were less likely to be embedded and sustained over time. Barriers to supporting children’s social-emotional skills included a lack of time, large group size, lack of educator capability, motivation, confidence and training, high staff turnover, perceived lack of recognition of the role educators play in supporting social-emotional development, and inconsistency in pedagogy and practice across services. Participants also perceived an increased proportion of children attending ECEC services with additional (both diagnosed and undiagnosed) needs, and requested greater support to nurture the diverse learning outcomes of children attending early learning programs.

Finally, educators sought programs that respond to the unique context and requirements of ECEC, aligned with the National Quality Standard and EYLF, and not requiring additional time or resources to implement. That is, resources that were accessible, easy to use, and could be embedded into daily practice and routines. Up-skilling educators in practical strategies and techniques that foster SEL was suggested by several participants, who noted that tools should respond to the different ways educators build knowledge. Coaching and mentoring were highlighted as effective in building capability within ECEC classrooms, and increased opportunity to reflect, collaborate, and share knowledge with team members was suggested.

#### 3.1.4. Feedback from the Advisory Group

The intervention design group similarly emphasised that an add-on program (i.e., curriculum-based SEL intervention) would likely encounter significant barriers to implementation. The group stressed: (i) programs that are not embedded within the classroom routines and aligned to the National Quality Standard and EYLF are unlikely to be sustained over time; (ii) each interaction in the room presents an opportunity to strengthen children’s social and emotional development; and (iii) educators who are confident in this role can assist parents to consider and implement strategies that will encourage social and emotional skills the home environment.

#### 3.1.5. Program Goal and Logic Model

**Figure 1:**
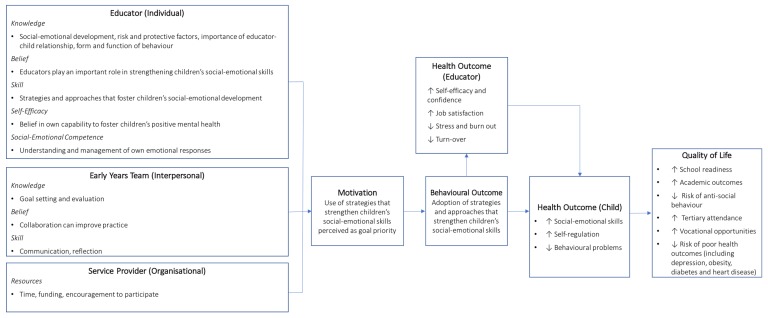
Logic model for Cheshire SEED Educational Program. SEED: Social-Emotional Engagement and Development.

The overall goal of Cheshire SEED was to improve children’s mental health in ECEC settings. Specifically, it sought to strengthen the everyday interactions between educators, children, and families so that early childhood educators could support and foster *all* children’s social and emotional development. The intervention design group decided Cheshire SEED would focus on the behaviour of the early childhood educator (at the individual level), and two environmental factors: educators’ peers (interpersonal level) and the ECEC service provider (organisational level). The needs assessment informed the development of a logic model (Figure 1), summarising the intervention levels (individual, interpersonal, and organisational), key determinants, behavioural outcome (use of strategies or approaches to support children’s social and emotional skills), and health outcomes for both the educator and child.

#### 3.1.6. A Framework to Guide Program Design

**Figure 2:**
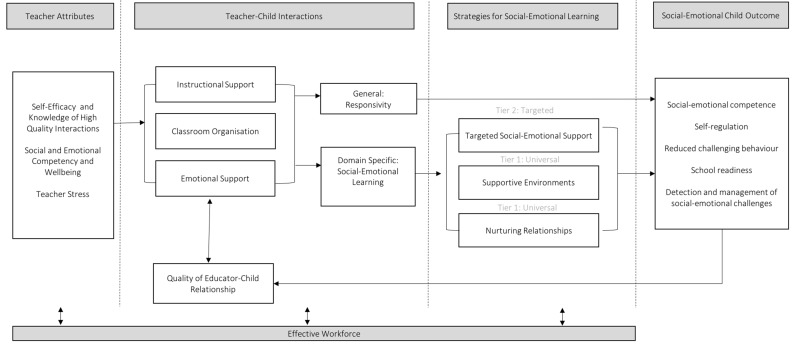
A Conceptual Model to Foster Social-Emotional Learning in Preschool Children by Targeting the Quality and Intentionality of Teacher–Child Interactions.

The program logic emphasised the change in educator behaviour (adoption of strategies to strengthen children’s social-emotional skills) to achieve the program goal (strengthening everyday interactions between educators, children and families). To further assist the intervention design process, a conceptual model was proposed (Figure 2) [73]. This model draws upon two frameworks that support educators to implement strategies to improve social-emotional development: the Teaching Through Interactions Framework [74] and the Pyramid Model for Supporting Social-Emotional Competence in Infants and Young Children [75,76]. It proposes embedding the intentional language, conversational techniques, and responsive practices that underpin high quality educator–child interactions within the framework of SEL strategies. As such, it aims to provide a roadmap for enhancing the quality and sustainability of the educator–child interactions critical in the social and emotional development of young children [73].

### 3.2. Step 2: Program Outcomes and Objectives

Performance objectives for each level of intervention (educator, educators’ peers, and ECEC service provider) are presented in Table 2. Educators’ knowledge, beliefs, skill, self-efficacy, and social and emotional competence were agreed as determinants of educator behaviour at the individual level (based on the findings from Step 1). At the interpersonal level, knowledge, beliefs, and skill were identified, and resources were the primary determinant at the organisational level. Change objectives at the individual educator level are provided in Table 3.

### 3.3. Step 3: Program Design

Informed by the outcomes of the systematic reviews and qualitative research, conceptual model and design workshops, the intervention design group proposed a multi-faceted learning tool for early childhood educators who want to build expertise in fostering children’s social and emotional skills. The following program components were discussed and prioritised during the design group workshops: a phone or tablet app of strategies, visual guides and factsheets, instructional bite size videos, professional learning community, educator workshops, and coaching at the point of practice. Following the workshops, the lead researcher reviewed behaviour change theories and methods suitable for the determinants and change objectives at each intervention level. Practical applications (specific activities) aligned with the behaviour change methods were identified (see Table 4 for strategies to achieve change objectives at the individual educator level). These approaches were embedded into the broad components identified by the design group.

### 3.4. Step 4: Program Production

Cheshire SEED was developed based on the preceding IM steps. SEED aims to build on educators’ knowledge by offering tailored, practical strategies for everyday practice that supports children’s social and emotional skills. The SEED Model could be utilised as a whole-room approach to encourage school readiness and positive mental health, or to plan an intervention for a particular child experiencing social, emotional, or behavioural challenges. The online learning program includes five sequential modules. Module 1 describes the program concepts and evidence that underpins the strategies and techniques. In Module 2, educators reflect on the strengths and challenges for children in their room, the factors that might be influencing behaviour using a Functional Behavioural Analysis approach (to identify when, where and the likely reason a behaviour occurs) [77], and set their goals for the program. Module 2 also incorporates content on social and emotional milestones, risk and protective factors, and form and function of behaviour. Based on the educator’s priorities in Module 2, Cheshire SEED suggests strategies in Modules 3 and 4 that may be particularly relevant for their group. Module 3 addresses the early learning environment, with a focus on sensory processing needs (e.g., layout, furniture, structuring the day, sensory tools and toys), and Module 4 on therapeutic and positive behaviour strategies that can be delivered through educator–child interactions.

The Cheshire SEED platform presents tailored content based on educator input. Each strategy includes an explanation of the technique and how it supports children’s development, a video explanation from the Cheshire School, step-by-step visual guide, examples of language and phrases, and an information sheet for caregivers. Several Module 3 strategies also include downloadable resources such as visual timetables and choice boards (graphic organiser that allows a child to show their choice), visual cards, and a feelings thermometer (a visual to help children identify the intensity of their feelings). Finally, Module 5 offers tools to assess whether the SEED strategies have benefited the children in the service. It also includes options to extend learning and share experiences with other educators using webinars and discussion boards.

### 3.5. Step 5: Program Implementation Plan

The Program Implementation Plan focused on mechanisms to deliver Cheshire SEED through ECEC providers and the ongoing support needed to ensure sustainability. It was decided the Lead Educator/Centre Director would facilitate implementation within their service, with support and guidance from the program provider. Each educator within a participating service creates an individualised profile to access Cheshire SEED. The next stage of this project will focus on child, educator, and process outcome measures for the intervention, and evaluating the feasibility and benefits of implementation across diverse early years settings.

## 4. Discussion

The aim of this paper is to describe the application of IM methodology to design, implement, and evaluate a pedagogical intervention to support positive mental health in preschoolers. A challenge for educational researchers is designing initiatives that are usable, sustainable and scalable [78]. While there has been growth in the availability of SEL programs for early years providers over recent decades [46,57,58,59,79,80,81,82], there is a paucity of literature that provides detail and transparency regarding design processes. To our knowledge, this is the first SEL program to use the IM approach, incorporating literature reviews, qualitative research with ECEC professionals, behaviour change theory, and co-design with early years and primary school educators, ECEC leaders, mental health professionals, and developmental researchers. Co-design across disciplines enabled us to address an important public health issue through the lens of early childhood, integrating health and education perspectives to break through the silos that can exist between disciplines and enhance the translation of health research to practice [83]. The application of IM to early childhood programming for social-emotional development appears to offer valuable insight to future researchers and program developers [48].

A challenge encountered during the design process was clearly articulating the opportunities for behaviour change in the Australian early childhood sector. ECEC services across Australia are diverse, with educators from varied educational backgrounds, with a range of qualifications, professional learning, and experiences. Early learning programs contrast in terms of their overall quality [84] and educators work with children and families with unique strengths and challenges [85,86]. The comprehensive nature of the needs assessment in Step 1 assisted the intervention design group to define program goals. Growing awareness of the lifelong implications of mental health in early childhood has seen a rapid increase in the availability of evidence-based interventions for ECEC providers. Four literature reviews indicated potential benefits of a tiered approach to SEL delivery [57,58,59], and highlighted the need for additional supports at the Tier 2 and 3 levels of intervention, especially for children showing signs of internalising behaviour.

The qualitative component of this work corroborated the need for practical and explicit strategies that built on educators’ current knowledge and expertise, could be embedded into their daily practice, and tailored to the social, emotional, and behavioural needs of the child. The intervention design group similarly suggested focusing on educators’ capability to promote SEL through their everyday interactions, by utilising the language, conversational strategies, and responsive practices that can support preschooler’s social-emotional competencies and learning outcomes. This finding was critical for the subsequent design of the program and underlines the importance of combining qualitative and quantitative data in this step.

The design group established both individual behavioural outcomes for educators, and environmental outcomes at the interpersonal and organisational levels (Table 2). During the qualitative research, educators emphasised they gain knowledge from their peers, and sought time and support to collaborate and share their knowledge with each other. The conceptual framework (Figure 2) highlighted the importance of workforce and systems to ensure continuity, effective training, and sustainability. The applications within Module 5 seek to address these interpersonal and environmental agents, including Communities of Practice and resources to assist providers to embed the program into their ongoing reflection, planning and systems.

Following the IM process ensured Cheshire SEED was a theory and evidence-based professional learning approach. Facilitating explicit knowledge is critical for educator learning [87], however studies suggest much educator knowledge is implicit and not articulated [88]. O’Connor and colleagues [89] found early childhood educators primarily drew on implicit knowledge, through observations and practical experience, to interpret parent-child relationships and children’s social and emotional development. They later developed the E-PCR program using the IM protocol to provide educators with knowledge and skills to first integrate implicit and explicit knowledge, and then translate this knowledge into their practice. Our qualitative research similarly explored how educators’ tacit knowledge influenced the strategies they use to strengthen children’s social and emotional skills. Building upon educators’ tacit knowledge by offering explicit, documented techniques could allow educators to integrate formal learning with personal experience. For example, an educator may already be using a Cheshire SEED technique in their professional practice. The SEED program offers an educator additional information about *why* that technique is valuable for children’s development by drawing on attachment theory, positive behaviour and support, play therapy, and positive psychology perspectives, thereby strengthening explicit knowledge.

The Cheshire SEED intervention was also strengthened by the co-design approach. While participatory design methods are commonly reported for health-related behaviour change interventions [90], this is an emerging methodology for educational curricula and reforms [91,92]. Collaborative processes that utilise skills, ideas, and experiences across disciplines are more likely to lead to change that is sustainable and scalable [93,94]. Cheshire SEED was directly shaped by the insights that emerged from participating educators, practitioners, and researchers.

There are also several limitations to the intervention. While a rationale for focusing on educator behaviour was provided, the caregiver and family environment are the first and foremost influence on children’s social and emotional skills. Cheshire SEED incorporates information that educators can provide to caregivers, however the intervention did not include influencing caregiver behaviour as an outcome [95]; this would require time and resource commitment beyond the scope of the project. In future research, it is recommended that consideration be given to incorporating the caregiver as an interpersonal level of intervention. In addition, it is vital that the future evaluation plan addresses both educator and child outcome measures. Our focus on educator behaviour seeks to ultimately improve child outcomes. Research indicates that strengthened educator–child interactions benefit children’s social, emotional and cognitive functioning [96], however the success of Cheshire SEED in achieving this goal is unknown. Informed by behaviour change theory, Cheshire SEED combines information on the risk and protective factors for social-emotional development with strategies that can be embedded into practice and pedagogy. The extent to which this approach is effective in addressing barriers such as educator self-efficacy also needs to be thoroughly evaluated (e.g., does this combination of information lead to increased confidence to change practice, or can it overwhelm participants?).

## 5. Conclusions

This paper describes the development of the Cheshire SEED Educational Program using the IM methodology. IM was successfully utilised to translate an evidence-based educational approach from an early primary school to early years setting. This was a comprehensive process that enabled a multi-disciplinary team to develop an intervention based on theory and evidence, with potential to be delivered at scale to early childhood educators. The findings suggest the IM protocol may offer a valuable roadmap for educators, educational researchers, and early childhood professionals to design interventions that target educator behaviour and practice.

## Figures and Tables

**Table 1:** Key Findings from Literature Reviews.

Type of SEL Program	Description of Review	Key Findings
Universal, curriculum-based SEL interventions [57]	Systematic review, meta-analysis and meta-regression of 79 experimental or quasi-experimental studies (391 effect sizes) that examined the impact of SEL intervention on children’s social, emotional, behavioural, and early learning outcomes	51 SEL programs examined. Children who participated in SEL programs showed significant improvement in social competence (*d* = 0.30, *p* < 0.001), emotional competence (*d* = 0.54, *p* < 0.001), behavioural self-regulation (*d* = 0.28, *p* < 0.001), and early learning skills (*d* = 0.18, *p* = 0.03), and reduced behavioural and emotional challenges (*d* = 0.19, *p* < 0.001). Older children appeared to display greater improvement than younger children, programs delivered by a researcher or specialist were more efficacious than those delivered by the educator, assessment of child outcomes based on educator, observer or researcher report indicated greater improvement than measures completed by caregivers, and children displayed greater improvement in skill-based measures, compared with educator, parent or observer rating.
Universal, curriculum-based SEL interventions	Systematic review of 16 studies (RCT, quasi-experimental, within-group designs) that examined the impact of SEL intervention on teaching quality and practice	10 SEL interventions examined. SEL programs may strengthen teaching quality, particularly the provision of emotional support, responsive and nurturing educator–child interactions, and effective management of the classroom environment. Data insufficient to ascertain whether participation improved educators’ knowledge, self-efficacy, or social-emotional wellbeing. No rigorous evidence of the sustainability of outcomes over time.
Tier 2 (targeted) SEL intervention [58]	Systematic review of 19 studies (RCT, quasi-experimental, single-subject designs) that examined the impact of Tier 2 SEL intervention on children’s social, emotional, and behavioural outcomes	Evidence for targeted SEL programming is emerging. May offer a promising early intervention approach to strengthen aspects of children’s social and behavioural functioning. Impact on emotional competencies could not be established. Programs directed to preschoolers with externalising problems, limited approaches focused on internalising behaviour.
Tier 3 (intensive) SEL intervention [59]	Narrative review of 19 studies (RCT, quasi-experimental, single-subject, within-group designs) that examined the impact of Tier 3 SEL intervention on children’s social, emotional, and behavioural outcomes	Interventions included instruction embedded into daily routines and activities, direct skill instruction, peer-mediated interventions, and individualised assessment-based approaches. Interventions targeted children with neurodevelopmental disorders, and developmental, social and communication delays. Improvement in children’s social skill during or post intervention. Evidence of maintenance and generalisation inconsistent. Lack of peer-reviewed research examining ECEC-based interventions for young children experiencing anxiety or mood disorders.

Note. SEL: social and emotional learning; RCT: Randomised Controlled Trial; ECEC: early childhood education and care.

**Table 2:** Program Outcomes and Performance Objectives for Cheshire SEED by Socio-Ecological Level.

Program Goal	Target Group	Program Outcome	Performance Objectives (PO)
To strengthen the everyday interactions between educators, children, and families so that early childhood educators can support and foster *all* children’s social and emotional development.	Educator (Individual)	Educators utilise strategies that target children’s social and emotional skill development during their everyday interactions and practice	*Educators will:* PO1: Develop nurturing, consistent, and responsive relationships with children PO2: Understand early childhood social, emotional, and behavioural development PO3: Identify the social-emotional strengths, challenges, and opportunities for children in their group PO4: Build knowledge of strategies, techniques, and language that supports young children’s social and emotional learning and positive mental health PO5: Respond effectively to opportunities to support social and emotional skill growth by applying strategies PO6: Engage with caregivers around strategies
	Peers/Early Years Team (Interpersonal)	Educators collaborate to establish goals, share knowledge and learning, and monitor progress	*Early Years Teams will:* PO7: Set goals for individual children and groups PO8: Encourage and support each other to implement strategies that target children’s social and emotional skill development P09: Reflect on any changes in children’s behaviour and social-emotional competencies as a result of strategies P10: Reflect on any changes in educators’ own practice as a result of strategies
	ECEC Service Providers (Organisational)	Service providers encourage ECEC staff to engage in professional development	*Service Providers will:* P11: Afford time and encouragement for educators to engage in learning, reflection and discussion, and embed strategies into their practice and routines

**Table 3:** Matrix of Change Objectives for Educators (Individual Level).

Educator Performance Objectives (PO)	Key Determinants				
	Knowledge (K)	Belief (B)	Skill (SK)	Self-Efficacy (SE)	Social-Emotional Competency (SO)
PO1: Develop nurturing, consistent, and responsive relationships with children	K1.1: Educators know how the educator–child relationship influences children’s behaviour and wellbeing K1.2: Educators understand factors that influence the educator–child relationship	B1.1: Recognise the importance of positive educator–child relationships for children’s mental health	SK1.1: Engage, interact, and respond sensitively to young children SK1.2 Share information and experiences through interactions SK1.3: Recognise, understand, and respond appropriately to social and emotional cues	SE1.1: Express confidence in ability to form positive relationships with children	SO1.1: Recognise own emotions and behaviour SO.1.2: Understand and manage own emotional responses
PO2: Understand early childhood social, emotional, and behavioural development	K2.1: Educators can describe social-emotional milestones that typically emerge in early childhood K2.2: Educators know the risk and protective factors for healthy social-emotional development K2.3: Educators can identify the outcomes associated with early social and emotional difficulties	B2.1: Recognise the importance of social and emotional competencies for learning, health, and wellbeing	SK2.1: Integrate knowledge gained through experience, professional development, and informal learning SK2.2: Build knowledge by working with peers and other professionals	SE2.1: Confidence in ability to gather, retain, and apply information	SO2.1: Recognise how own experiences, background, and culture can influence understanding and perceptions of child development
PO 3: Identify the social-emotional strengths, challenges, and opportunities for children in their group	K3.1: Educators recognise behaviours that suggest healthy social-emotional development K3.2: Educators can describe common form (types) of challenging behaviours K3.3: Educators can describe possible functions (purpose) of behaviour	B3.1: Belief that ECEC educators play an important role in observing and understanding child behaviour	SK3.1: Identify form and function of behaviours SK3.2: Collect and interpret information from different sources (e.g., observation, caregiver, other early years professionals)	SE.3.1: Belief in ability to understand and respond to children’s behaviour	SO3.1: Recognise how own experiences, background, and culture can influence perception child behaviour
PO4: Build knowledge of strategies, techniques, and language that supports young children’s social and emotional learning	K4.1: Educator knows how the early years environment, caregiver–child, and child–child interactions can influence social-emotional development K4.2: Educators understand theories and principles that underpin strategies K4.3: Educators understand the purpose and rationale of strategies K4.4: Educators know how to use the strategy effectively	B4.1: Perceive ECEC educator is responsible for supporting social-emotional skill development B4.2: Recognise educator–child interactions can have therapeutic benefit B4.3: Recognise early years environment can influence children’s social and emotional skill B4.4: Belief that strategies can build upon educators’ current skill and knowledge	SK4.1: Integrate new knowledge (strategies) with current knowledge and practice	SE4.1: Express confidence in ability to use strategies during every day practice	
PO5: Respond effectively to opportunities to support social and emotional skill growth by applying strategies	K5.1: Educator can identify suitable strategies based on needs and challenges of child/group	B5.1: Increased recognition that every interaction is an opportunity to nurture children’s social and emotional skill	SK5.1: Identify opportunities to embed strategies into daily interactions and practice SK5.2: Implement strategies	SE5.1: Belief in ability to implement strategies	SO5.1: Recognise how own experiences, background. and culture can influence interactions with children
PO6: Engage with caregivers around strategies	K6.1: Educator can describe approaches that strengthen children’s social-emotional skills	B6.1: Belief that educator and caregiver should work in partnership to support children’s social-emotional development	SK6.1: Ability to engage caregivers in conversation about their child’s development SK6.2: Ability to share and discuss strategies	SE6.1: Confidence in ability to work in partnership with caregivers	SO6.1: Recognise how own experiences, background, and culture can influence interactions with caregivers and families

**Table 4:** Examples of Strategies to Achieve Change Objectives for Educators.

Level of Intervention	Determinant of Educator Behaviour	Change Objective (s)	Method (Related Theory)	Specific Activities in Cheshire SEED
Educator (Individual)	Knowledge	K1.1, 1.2, 2.1, 2,2, 2.3, 3.1, 3.2, 3.3, 4.1, 4.2, 4.3, 4.4, 6.1	Active Learning (SCT, SLT, ELM)	Interactive modules Goal setting, observation, and reflection Interactive case studies
		K1.1, 1.2, 3.1, 3.2, 3.3, 4.1	Consciousness Raising (TTM)	Written and video content
		K5.1	Tailoring (TTM)	Tailored SEL strategies based on user inputs
		K4.1, 4.2, 4.3, 4.4, 5.1	Discussion (ELM)	Moderated online communities of practice forums Webinar In-room consultation
	Belief	B1.1, 2.1, 3.1, 4.1, 4.2, 4.3, 4.4, 5.1, 6.1,	Elaboration (TIP, ELM)	SEL strategies Video by coaches
		B3.1, 4.1, 4.2, 4.3, 4.4, 5.1	Argument/Persuasive Communication (ELM, TPC)	Video by coaches
		B4.2, 4.3, 4.4, 5.1	Direct Experience (TL)	SEL strategies In-room consultation
	Skill	SK1.1, 1.2, 1.3, 2.1, 2.2, 3.1, 3.2, 4.1, 5.1, 5.2, 6.1, 6.2	Active Learning (SCT, SLT, ELM)	Interactive modules Goal setting, observation, and reflection Interactive case studies Parent handouts
		SK5.1, 5.2, 6.1, 6.2	Individualisation (TTM)	In-room consultation Communities of practice forums Webinars
		SK1.1, 1.2, 1.3, 3.1, 5.1, 5.2	Verbal Persuasion (SCT)	Video by coaches
		SK5.1	Goal Setting (TSR)	Goal setting, observation, and reflection
		SK3.1, 4.1, 5.1, 5.2	Modelling (SCT)	Video exemplars Examples of language and phrases In-room consultation Case studies
		SK3.1, 3.2, 4.1, 5.1, 5.2	Participatory Problem Solving	Functional Behaviour Analysis Individualised plans
	Self-Efficacy	SE1.1, 3.1, 4.1, 5.1	Guided Practice and Feedback (SCT, TSR)	In-room consultation
		SE1.1, 2.1, 3.1, 4.1, 5.1, 6.1	Discussion (ELM)	Communities of practice forums Webinars
	Social-Emotional Competence	SO1.1, 1.2, 2.1, 3.1, 5.1, 6.1	Guided Practice and Feedback (SCT)	In-room consultation
		SO1.1, 1.2, 2.1, 3.1, 5.1, 6.1	Consciousness Raising (TTM)	Written and video content

Note. ELM, Elaboration Likelihood Model; SCT, Social Cognitive Theory; SLT, Social Learning Theory; TPC, Theories of Persuasive Communication; TTM, Trans Theoretical Model; TIP, Theories of Information Processing; TL, Theories of Learning; TSR, Theories of Self-Regulation.
